# Public-private partnerships are critical for rapid response to infectious disease threats

**DOI:** 10.3389/fpubh.2025.1695424

**Published:** 2026-01-14

**Authors:** Jennifer Rakeman-Cagno, Larry Kelmar, Michael Loeffelholz, David Persing

**Affiliations:** 1 Cepheid, Medical and Scientific Affairs, Public Health Programs, Sunnyvale, CA, United States; 2 Cepheid, Government Programs and Pharma Alliances, Sunnyvale, CA, United States; 3 Cepheid, Medical and Scientific Affairs, Sunnyvale, CA, United States

**Keywords:** diagnostic testing, outbreak response, pandemic preparedness, public health emergency response, public-private partnerships

## Abstract

Public-private partnerships focused on infectious disease diagnostics have been increasing since the COVID-19 pandemic. These partnerships have resulted in new test development, increased testing capacity and services, technology development, and processes to enable faster collaborative response in the context of an outbreak. This paper explores the importance of public-private partnerships in response to infectious disease public health threats. Collaboration between federal partners and diagnostic test manufacturers has been critical to the COVID-19, mpox, and other responses in the United States, and these partnerships will be critical to future responses. Public-private partnerships pull together the pieces needed to rapidly develop and scale diagnostics necessary for responding to emerging infectious diseases. Developing partnerships during “peace time” further enables rapid action when outbreaks occur.

## 1 Introduction

Public-private partnerships are essential for the early detection and rapid response to biothreats and emerging infectious agents. In the United States, partnerships between federal agencies and diagnostic test manufacturers have in several cases resulted in rapid development and deployment of tests essential for public health response. However, in the early months of the COVID-19 pandemic, multiple issues led to the lack of adequate access to diagnostic testing which significantly impeded the response in the United States (1). In this paper, we explore examples of public-private partnerships in the US that aim to address the diagnostic gaps that were recognized in the COVID-19 response. In addition to these federal partnerships, the relationships between diagnostic test manufacturers and state/local/tribal health departments and public health laboratories help to ensure that these critical voices are heard and public health needs are addressed as diagnostic tests are developed and deployed. Partnerships ensure that tests in development are held to consistent standards of performance, that tests will be available on multiple platforms, are available for use in appropriate settings, and are equitably accessible.

Some of the biggest challenges faced by diagnostic manufacturers to develop tests to address emerging pathogens of public health significance include lack of access to data (including sequence data), samples, and reagents (including reference material that can be transported to and handled in laboratories safely) to develop and validate tests, and regulatory timelines that are not compatible with public health emergency (PHE) response. Importantly, manufacturers often have to wrestle with lack of a business case that supports assignment of company resources to an intensive and costly test development project in the face of uncertain demand.

In these collaborations, government partners offer funding, research and development (R&D), access to key subject matter experts and data, and access to critical materials (including control materials and specimens) and reagents. The industry partner contributes by offering R&D expertise, development of tests on existing platforms with an established installed base, and by providing rapid scalability in manufacturing and distribution. These partnerships also enable real time collaborative links with other government partners, including the Food and Drug Administration (FDA), which can accelerate the regulatory approval processes while ensuring that quality standards are upheld.

## 2 Pathogen emergence to pandemic response

**Figure 1:**
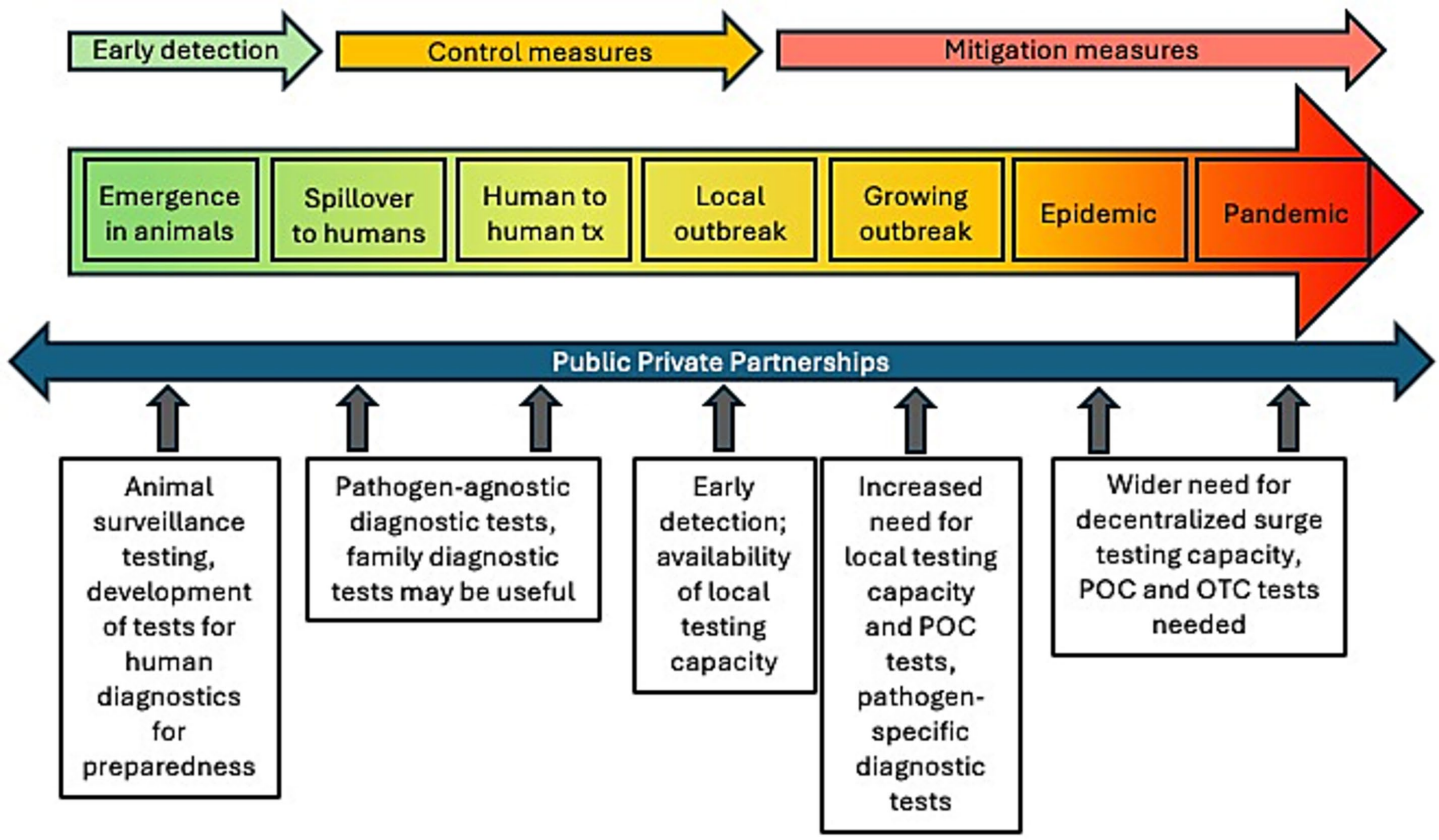
Public-private partnership use cases at each phase of an infectious disease public health emergency. tx, transmission; POC, point-of-care; OTC, over-the-counter.

Infectious disease emergencies caused by pathogens for which there are not existing diagnostic tests, vaccines, and/or treatments have generally arisen as zoonoses (Figure 1). The disease initially emerges in animals, and spillover occurs when infected animals (or contaminated environments or arthropod vectors) and humans are in close contact. If subsequent human to human transmission occurs, there is significant threat of an outbreak in humans.

Surveillance for and detection of these events is achieved through existing public health surveillance programs led by the CDC. These surveillance programs depend on funding and a strong public health laboratory system in the US and globally to successfully detect animal outbreaks, spillover events, and potential human outbreaks.

Once a spillover event has been detected and there is threat of further spread, CDC and state/local/tribal health departments send health alerts to clinicians to provide information on clinical and epidemiological characteristics of potential cases. Health departments investigate cases and clusters, and laboratories implement tests to diagnose cases (and rule out pathogen presence in uninfected patients). Early on, diagnostic tests may not be readily available in clinical labs and specimens may need to be sent to state or local public health laboratories or the CDC for diagnostic testing. This limits testing capacity and prolongs time to actionable results, especially during periods of high demand. During the control phase of an outbreak, having adequate diagnostic capacity locally available is critical to detect cases, test contacts, and implement public health control measures to stop the spread. Rapid electronic reporting of diagnostic test results to public health authorities is important for decision making and implementation of public health measures. Controlling and stopping an outbreak at this early stage depends on the availability of tests to identify cases and inform the implementation of public health measures such as isolation of cases, treatments and/or vaccines.

If the outbreak grows and spreads (potentially becoming an epidemic or pandemic), the public health response will shift from control to mitigation. In this phase, the strategy is focused on reducing the impact of the disease. Surveillance and diagnostic testing continue, and the need for high volumes of readily available testing becomes critical. Public health interventions will be informed by case counts and continued monitoring of the epidemiology of the public health emergency (PHE).

Quickly developing, manufacturing, and deploying tests (as well as medication and vaccines) to where they are needed requires public-private partnerships. The challenge lies in developing diagnostic tests ready for wide deployment when there is little known about the pathogen, few cases (and therefore few samples available), no available pathogen-specific reagents, and an unknown return on investment for the development of a test. Traditional regulatory pathways to bring a test to market take too long to be responsive to an emerging pathogen.

## 3 Key collaborations

In the US, key partners include the National Institutes of Health (NIH) Independent Test Assessment Program (ITAP), the Centers for Disease Control and Prevention (CDC), the Biomedical Advanced Research and Development Authority (BARDA), and the Department of Defense (DoD), as well as private diagnostic test manufacturers. These partnerships have led to significant advancements that include rapid response to outbreaks, building infrastructure to meet manufacturing and distribution needs for response, and preparing for future outbreaks. The partnerships help to ensure that quality diagnostic tests are available when and where they are needed to inform public health measures for outbreak control and mitigation.

ITAP was established in 2021 by the NIH under the leadership of Dr. Francis Collins to create an accelerated pathway for the FDA to evaluate at-home COVID-19 tests as part of the national response to the pandemic. This pathway enabled the regulatory agency to assess the performance of the tests and authorize tests quickly for rapid scale-up in manufacturing. The program was successful in bringing multiple quality at-home diagnostic tests to the market to meet the needs of the pandemic response (2).

Since then, the National Institute of Biomedical Imaging and Bioengineering (NIBIB), home to the Rapid Acceleration of Diagnostics Technology (RADx Tech) program and ITAP, has built upon the success of the model to address other emergency responses and diseases. Projects have included development and deployment of point of care and lab-based tests for mpox, development of a lesion panel that includes mpox, development and deployment of hepatitis C and hepatitis B point of care diagnostics to support US HCV elimination efforts, and the development of HIV point of care viral load tests (3).

A notable example is the ITAP-Cepheid mpox partnership, in which a molecular test for mpox was developed and received an Emergency Use Authorization (EUA) in less than 6 months from the section 564 declaration (by the Secretary of Health and Human Services which opens the FDA EUA pathway) (4). The test is authorized for use in point of care (POC) (CLIA-waived) and laboratory settings, making rapid diagnosis of new cases and implementation of treatment and prevention measures possible in a single patient visit. The partnership included support for some of the studies required for FDA authorization, access to samples for analytical and clinical studies, and importantly, the ability to move through the regulatory process quickly. The ITAP team was able to communicate information between the manufacturer and FDA, and the regulatory submission was done in sections as they were ready, which sped up the review process. (In 2024, the World Health Organization (WHO) listed the test under its Emergency Use Listing (EUL) procedure, and the test has been used globally for outbreak response.) (5) Without access to specimens, support for analytical and clinical studies, and strategic collaboration between partners, this accelerated timeline would not have been possible. In addition, the public investment in the project made it feasible from a business perspective.

This project also created a collaborative relationship between the public and private entities involved. Throughout the process, the challenges, strengths, and points of view of the partners were clear and deepened understanding across the sectors. When ITAP and Cepheid later partnered to develop a hepatitis C point of care test for the US, the relationships were already established. Over a matter of months, the test was developed, validated, and received *de novo* authorization for use in POC (CLIA-waived) settings (6).

CDC surveillance programs are able to detect and characterize emerging pathogens, but the agency is not able to provide the diagnostic capacity and fast turnaround times for result reporting needed as outbreaks develop. Partnerships with CDC and diagnostic manufacturers are important – industry can scale up manufacturing and distribution quickly but need partnerships with CDC for access to subject matter expertise, data, samples, and reagents. Recently, CDC issued a solicitation that will result in formalized contracts with diagnostic test manufacturers for the development and production of diagnostic tests for emergency response (7). These contracts would allow partnerships to move forward very quickly in a PHE because the agreements and plans to move forward on data, expertise, sample and reagent sharing, test development, regulatory authorization, manufacturing, and distribution would already be in place.

CDC has also partnered with commercial laboratories to increase test capacity and reduce test turnaround times. When Zika testing needs outpaced availability for testing at public health laboratories, CDC partnered with commercial laboratories to increase testing capacity. The relationships built during this outbreak became the foundation for further collaboration. (8) During the US mpox outbreak, CDC collaborated with a number of commercial laboratories to increase testing capacity by enabling these labs to implement their CDC developed test on high throughput reference laboratory platforms. This collaboration resulted in an increase of the national testing capacity by 10-fold, decreased test turn-around times, and shifted significant mpox testing burden away from public health laboratories that were already stretched thin. (9) In 2024, CDC awarded contracts with six entities to support increased ability to respond to public health threats by providing access to testing at scale and to enhance access to data to support surveillance and situational awareness. (10).

The Detection, Diagnostics, and Devices Infrastructure (DDDI) Division at BARDA focuses on rapid response to emerging infectious threats, pandemic influenza, and CBRN (chemical, biological, radiological and nuclear) threats. The Division has provided funding and support to diagnostic manufacturers for projects that have resulted in FDA clearance/authorization for 42 diagnostic devices to address public health needs including outbreak/pandemic detection and response between 2011 and 2025 (11). The Diagnostic Rapid Response (DxR2) initiative was launched in March 2024. The aim of this BARDA program is to work with diagnostic manufacturers to build a test portfolio for biothreat agents that can be manufactured and deployed at scale just in time.

BARDA-funded projects focus on outbreak readiness and response, including support for the development and regulatory clearance of tests to detect influenza viruses with pandemic potential, pathogen-agnostic diagnostics, pathogen family-based diagnostics, and detection of antimicrobial resistance. Strategically, the focus is on early detection and early response to emerging pathogens and the goal is to have diagnostic tests ready to deploy before the initial cases are detected.

The DoD collaborates with diagnostic manufacturers to support the National Biodefense Strategy by developing tests for emerging threats through the Joint Program Executive Office (JPEO). Partnerships with diagnostic manufacturers to develop diagnostic tests for military use meets DoD needs and results in advancement of technology and development of tests that could be deployed for civilian use in a PHE. Man-Portable Diagnostic Systems (MPDS) are developed to be used in far-forward environments under harsh environmental conditions, and these technologies may be leveraged for decentralized testing (12).

Diagnostic tests that are developed through any of these programs need to be deployable, function within existing and future workflows and testing settings, be equitably accessible to the public, and the data from these tests need to be reported to public health agencies. Partnerships with CDC, the Association for Public Health Laboratories (APHL), and others is critical to ensure that tests and the data they generate are compatible with data reporting systems and that there are mechanisms to report the data. This remains a challenge especially in the context of widespread use of POC and over the counter (OTC)/home based testing. Continued partnerships and support of programs like CDC’s Data Modernization Initiative (DMI) are essential (13).

## 4 Discussion

Public-private partnerships are a critical component of the response to an infectious disease threat to public health. These collaborations are essential for the rapid development, deployment, and scale up of diagnostic tests needed for an effective outbreak response.

For effective response in the early phases of emergence of a pathogen, diagnostic tests must be ready and deployable, but for commercial test developers, the decision to deploy internal resources before tests are needed can be difficult without any guarantee of return on investment. Programs funded by BARDA address this gap by providing resources for development and validation of tests with no immediate use case. These collaborations provide access to data and samples to diagnostic manufacturers as well as funding to offset the costs, without which these tests would not be developed.

Once a pathogen has been identified and characterized and spillover human cases are occurring, programs like the NIH RADx and ITAP programs become critical to getting specific diagnostic tests that can be deployed at scale in a decentralized manner through development and the regulatory process rapidly. Collaborative strategizing and access to answers with rapid turnaround make this program successful.

In addition, CDC partnerships with commercial labs and diagnostic manufacturers can amplify testing capacity and access using the commercial lab and industry infrastructure to scale. CDC supplies information, reagents, data, R&D, and prototypes that can be further developed by private partners into tests to be manufactured and widely distributed and/or made available via high throughput testing labs. CDC also continues to work across sectors to make data reporting and real-time data sharing feasible during public health emergencies.

As outbreaks grow and the response moves into the mitigation phase, low-threshold access to testing becomes increasingly important. When the COVID-19 response was in this phase, ITAP worked with manufacturers to ensure quality tests were available at scale in the POC and over the counter (home testing) spaces. The Xpert Mpox test, developed through public-private partnership, was deployed in laboratory and decentralized POC settings in the US during the 2022 outbreak. The test continues to be used to aid in the diagnosis of sporadic US cases, enabling rapid public health follow up. In 2024, the test was used in highly decentralized settings in response to outbreaks in multiple African countries (14). In 2015, the Xpert Ebola test was deployed by the Liberian Ministry of Health with support from public and private entities on a mobile laboratory to support enhanced surveillance activities in areas where testing was not otherwise readily available (15). Ongoing collaborations between federal partners and industry will ensure that tests are available, that they evolve with the pathogen (for example, able to detect emerging variants), and that the data generated through testing is appropriately reported to public health authorities.

Here, we focused on public-private partnerships between government and industry. There also needs to be on-going collaboration and dialogue between diagnostic manufacturers, clinical laboratories, public health laboratories, and academia (16). The voices of clinical labs, public health labs, and health departments are critical to ensure the tools developed fit workflows and generate the data necessary for public health response decision making and action. Rapid communication, collaboration, and support allows for tests and testing capacity necessary for a PHE response to get to market faster. Programs like ITAP also invest in assessment of test performance to ensure tests provide actionable results. Public investments help to ensure that tests that would otherwise lack financial justification are supported and maximize patient impact.

Public-private partnerships have been instrumental in the responses to multiple infectious disease threats. These partnerships have enabled the rapid development of diagnostic tests, deployment of tests to critical areas around the globe, and have advanced the development of new technologies. Public-private partnerships need to be on-going. “Peace time” collaboration enables rapid action when outbreaks occur. These relationships are essential to rapid detection and robust response to ever-growing infectious disease public health threats. Without close collaboration across public and private sectors, the development and deployment at scale of tests critical to the detection, monitoring, and response to PHE will lag, and the tools necessary to avoid the impact of a pandemic like COVID-19 will not be ready when we need them.

## Data Availability

The original contributions presented in the study are included in the article/supplementary material, further inquiries can be directed to the corresponding author.
